# Reactive oxygen species and antioxidant strategies in human sperm cryodamage

**DOI:** 10.3389/fmed.2026.1870054

**Published:** 2026-08-17

**Authors:** Luca Tramontano, Roberto Marci, Romualdo Sciorio, Jean-Marie Wenger

**Affiliations:** 1 Réseau Hospitalier Neuchâtelois (RHNE), Neuchâtel, Switzerland; 2 Hôpital de La Tour, Meyrin, Switzerland; 3 Università degli Studi di Ferrara, Ferrara, Italy; 4 Vitrolife Sweden AB, Västra Frölunda, Sweden

**Keywords:** antioxidants, DNA fragmentation, male fertility, oxidative stress, reactive oxygen species, sperm cryopreservation, spermatozoa

## Abstract

Sperm cryopreservation is widely used for male fertility preservation and assisted reproduction technologies; however, the freezing and thawing process can induce irreversible structural and functional damage to human spermatozoa. This review summarizes the main mechanisms through which cryopreservation disrupts redox homeostasis, with particular emphasis on the generation of reactive oxygen species, mitochondrial dysfunction, lipid peroxidation, DNA fragmentation, and the consequent impairment of sperm motility and viability. Human spermatozoa are particularly vulnerable to oxidative damage because they possess limited cytoplasmic antioxidant defenses and a plasma membrane rich in polyunsaturated fatty acids (PFA), which are highly susceptible to peroxidative injury. In addition, the dilution or removal of seminal plasma during cryopreservation further compromise extracellular antioxidant protection. Experimental studies have shown that supplementation of freezing and/or thawing media with enzymatic and non-enzymatic antioxidants, including catalase, glutathione, ascorbic acid, tocopherols, melatonin, resveratrol, quercetin, MitoTEMPO, myo-inositol, elamipretide, brain-derived neurotrophic factor, and various natural extracts, may improve selected post-thaw sperm parameters and reduce biomarkers of oxidative stress. Nevertheless, no antioxidant strategy currently available is capable of completely preventing cryodamage, and the market heterogeneity of existing protocols limits their clinical translation. Further well-designed human studies are therefore required to determine the safety, efficacy, optimal concentration, and clinical utility of antioxidant supplementation in sperm cryopreservation media.

## Introduction

Sperm cryopreservation is a widely used and effective technique for preserving male fertility in patients at risk of impaired sperm quality or testicular function. It is commonly applied before treatments such as chemotherapy, radiotherapy, and surgery, as well as in cases of severe oligozoospermia (1–3). In assisted reproductive technology (ART), cryopreservation is routinely used for donor semen storage and for preserving spermatozoa retrieved from azoospermic patients undergoing testicular sperm extraction (4, 5). Despite continuous improvements in cryopreservation protocols, several technical and biological challenges remain unresolved. The process of cooling, freezing, and thawing can significantly reduce sperm survival and negatively affect sperm function. These procedures may alter membrane lipid composition and acrosome integrity, while also increasing DNA fragmentation and oxidative damage (6). In addition, cryopreservation often decreases sperm motility and viability, compromising overall sperm quality. The main causes of cryodamage include osmotic stress, cold shock, intracellular ice crystal formation, excessive generation of reactive oxygen species (ROS), and disruption in antioxidant defense mechanisms (4, 7). These factors can activate apoptotic pathways and lipid peroxidation, leading to impaired sperm motility, viability, mitochondrial activity, chromatin integrity, and membrane stability (8–11). Consequently, although sperm cryopreservation remains essential for fertility preservation and ART, further research is needed to minimize cryoinjury and improve post-thaw sperm quality. Spermatozoa from ejaculates with poor baseline semen quality appear particularly vulnerable; the recovery of motile and viable spermatozoa after freeze–thaw procedures is reduced when even one basal semen parameter is below the fifth percentile of the World Health Organization reference values (6). Table 1 summarizes those references. Over the past decades, numerous strategies have been performed in different mammalian species to reduce damage associated with sperm cryopreservation. Freezing and thawing procedures, expose spermatozoa to severe physical and biochemical stress; thus, several approaches have been tried on limiting cold shock, ice crystal formation, and oxidative injury. Among these strategies, increasing membrane fluidity has been proposed to improve sperm resistance to low temperatures and to preserve membrane function and integrity during cooling. Also, cryoprotectant agents have been applied to reduce heterogeneous ice nucleation and intracellular ice crystallization, both of which contribute significantly to cellular injury during the freeze–thaw process. Another area investigated has been the reduction of ROS generation during semen processing, with an effort made to reduce major ROS sources, including leukocytes, or dead and defective spermatozoa. A largebody of literature has investigated the molecular pathways activated by freeze–thaw stress and has evaluated the potential protective role of antioxidant supplementation. Both enzymatic and non-enzymatic antioxidants, as well as plant-derived compounds and reducing agents, have been tested with the aim of scavenging excess ROS and preserving sperm function during cryopreservation (12). Experimental studies have reported beneficial effects of several antioxidants on post-thaw sperm quality, including improvements in motility, membrane integrity, mitochondrial activity, and DNA stability. However, most of this evidence comes from animal species. As a consequence, the evidence supporting the manipulation of sperm-freezing media to minimize oxidative damage in humans remains less consistent (13). Human spermatozoa present unique biological characteristics that may influence their susceptibility to cryoinjury, including limited endogenous antioxidant defences and a plasma membrane highly enriched in polyunsaturated fatty acids (PFA), which are particularly vulnerable to lipid peroxidation. For this reason, a better understanding of the mechanisms underlying cryodamage is therefore essential to optimize cryopreservation protocols and improve the quality and functional competence of thawed human sperm samples (14, 15). In this context, the present review summarizes the role of ROS in cryopreservation-induced sperm damage and critically examines the antioxidant strategies that have been investigated to counteract oxidative stress (OS) and preserve human sperm function during freezing and thawing procedures.

**Table 1:** This table presents the reference limits for sperm assessment parameters in semen analysis according to the World Health Organization Laboratory Manual for the Examination and Processing of Human Semen, 6th Edition (2021).

Parameter	WHO 6^th^ Edition lower reference limit
Semen volume	1.4 mL
Total sperm number	39 million/ejaculate
Sperm concentration	16 million/mL
Total motility	42%
Progressive motility	30%
Vitality	54%
Normal morphology	4%

## Reactive oxygen species and antioxidant systems in human sperm

**Figure 1:**
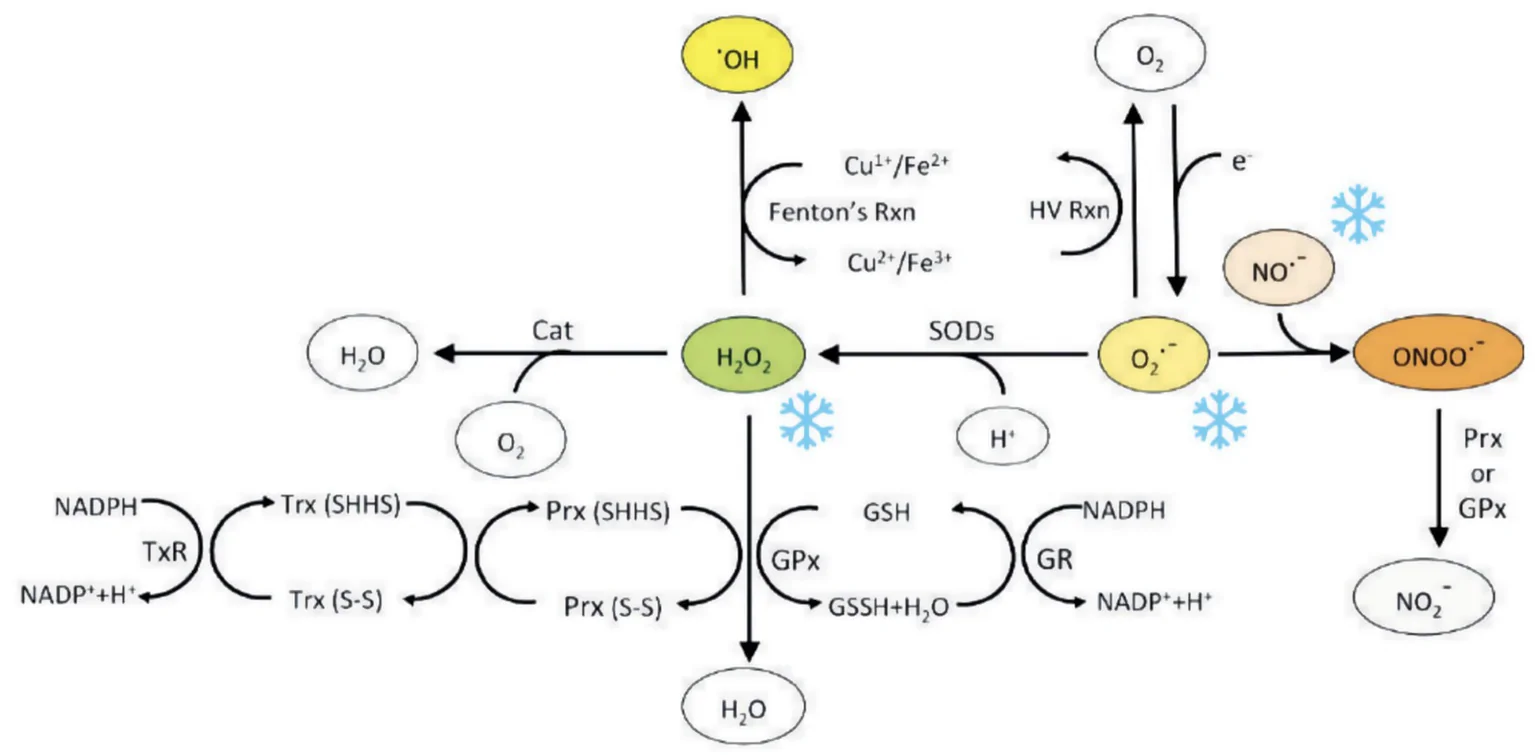
Schematic overview of reactive oxygen and nitrogen species generation and detoxification pathways during sperm cryopreservation, showing superoxide, hydrogen peroxide, and hydroxyl radicals, as well as antioxidant enzymes involved in intracellular antioxidants defense, including superoxide dismutase, catalase, glutathione peroxidase, glutathione reductase, peroxiredoxins, and thioredoxins. Snowflakes indicate reactive oxygen species generated during cryopreservation procedures.

Reactive oxygen species play a central role in sperm physiology and are required at low concentrations for essential reproductive processes, including sperm capacitation, the acrosome reaction, hyperactivation, and binding to the zona pellucida (16, 17). ROS comprise both free-radical and non-free-radical oxygen-derived reactive molecules generated mainly through the mitochondrial electron transport chain and several enzymatic reactions. The most common ROS include the superoxide anion, hydrogen peroxide, singlet oxygen, and hydroxyl radicals (Table 2). Under physiological conditions, controlled ROS production contributes to intracellular signaling pathways involved in fertilization. However, excessive ROS generation disrupts redox balance and promotes OS, leading to lipid peroxidation, mitochondrial dysfunction, DNA fragmentation, and reduced sperm motility and viability. Superoxide anions can undergo spontaneous or enzyme-mediated dismutation to form hydrogen peroxide. This compound can diffuse across cellular compartments and thereby contribute to long-range redox signaling. Hydrogen peroxide can also react with selected biomolecules; with the presence of ferrous iron, hydrogen peroxide can undergo the Fenton reaction, generating highly reactive hydroxyl radicals capable of damaging proteins, lipids and nucleic acids. To counteract oxidative damage, sperm cells rely on antioxidant defense systems, including superoxide dismutase, catalase, glutathione peroxidase/glutathione reductase and peroxiredoxin/thioredoxin pathways, which use reduced nicotinamide adenine dinucleotide phosphate as a reducing equivalent to convert hydrogen peroxide to water (Figure 1). Reactive nitrogen species, particularly nitric oxide and peroxynitrite, play a relevant role in sperm redox biology by regulating cellular signaling pathways, influencing sperm motility and capacitation, and contributing to oxidative balance, although excessive levels may also induce OS and impair sperm function (18, 19). Under physiological conditions, spermatozoa produce low amounts of ROS, primarily as a byproduct of mitochondrial activity during cellular respiration, where these molecules participate in normal signaling pathways that support sperm motility and other essential reproductive functions (16, 20). In contrast, excessive levels of ROS in semen may arise from immature germ cells and activated leukocytes, leading to OS that adversely affects sperm membrane integrity, DNA stability, and overall sperm function, thus reducing male fertility potential and reproductive outcomes (21, 22). Spermatozoa are highly susceptible to oxidative damage due to their limited intracellular antioxidant defenses and the high concentration of PFA present within their plasma membrane (16, 22, 23). These fatty acids are essential for maintaining membrane flexibility, supporting progressive sperm motility, and facilitating critical fertilization processes such as the acrosome reaction and fusion between sperm and oocyte membranes. However, PFA are extremely sensitive to ROS and free-radical attack, and excessive OS can easily trigger lipid peroxidation, ultimately impairing sperm structure, function, and fertilizing capacity (20, 24, 25). Peroxidation of membrane PFA initiates a cascade of lipid peroxidation at the sperm surface, disrupting membrane permeability and fluidity and leading to irreversible loss of motility and impaired sperm-oocyte fusion (16). To protect against excessive production of ROS, spermatozoa together with seminal plasma are equipped with both enzymatic and non-enzymatic antioxidant defense systems, which are particularly important at the post-testicular level during epididymal maturation and ejaculation (14, 16). The principal antioxidant enzymes present in seminal plasma include superoxide dismutase, catalase and glutathione peroxidase (26). Cytosolic copper-zinc superoxide dismutase and mitochondrial manganese-dependent superoxide dismutase catalyze the conversion of superoxide anions into hydrogen peroxide. Subsequently, hydrogen peroxide is detoxified and converted into water by catalase and glutathione peroxidase (Figure 1). In this process, reduced glutathione is oxidized to glutathione disulfide, which is then recycled back to its reduced form by glutathione reductase using nicotinamide adenine dinucleotide phosphate (NADPH), thereby maintaining redox homeostasis and protecting sperm function (18). Seminal plasma also contains a wide range of non-enzymatic antioxidants that play a crucial role in protecting sperm cells from oxidative damage. These antioxidants include ascorbic acid, alpha-tocopherol, pyruvate, glutathione, L-carnitine, taurine, hypotaurine, flavonoids, alkaloids, carotenoids, urate, albumin and ubiquinol (17, 26). Together, they help neutralize ROS and maintain sperm function and viability. Studies have shown that infertile men often exhibit reduced seminal antioxidant capacity associated with elevated ROS levels in their ejaculates (17, 26–28). OS is widely recognized as a major contributor to sperm dysfunction and DNA damage. However, it remains unclear whether increased ROS levels result primarily from excessive production, impaired antioxidant scavenging activity, or a combination of both mechanisms (17, 26).

**Table 2:** Table summarizing the effects of different antioxidants on sperm quality and oxidative stress parameters during cryopreservation.

Antioxidant	Positive effects
Ascorbic acid (63, 74)	ROS (↓); viability and weak motility (↑); apoptotic cells (↓); DNA damage (↓)
BDNF (73)	Sperm motility and vitality (↑); H2O2 (↓); caspase activity (↓)
Brown algae Sargassum extracts (68)	ROS (↓); sperm motility (↑)
Catalase (74)	ROS (↓); sperm viability and weak motility (↑); MMP (↑); apoptotic cells (↓); DNA damage (↓)
L-carnitine (79)	Sperm viability and motility (↑)
Elamipretide (78)	Sperm motility and viability (↑); plasma membrane stability (↑); MMP (↑); acrosome dysfunction (↓)
Glutathione (75)	DNA damage (↓); MDA (↓); ROS (↓)
Melatonin (29, 59, 64)	Total antioxidant capacity (↑); GSH concentration (↑); mitochondrial membrane integrity (↑); MMP (↑); SOD, catalase and GPx activity (↑); BCL-2, SOD2, GSTM1, NRF2 and HSP90AA1 expression (↑); lipid peroxidation and ROS levels (↓); NADPH oxidase activity (↓); Nox5 and Bax expression (↓); sperm viability and motility (↑)
MitoTEMPO (76)	Sperm motility and vitality (↑); sperm membrane integrity (↑); MMP (↑); SOD, catalase and GPx activity (↑); MDA levels (↓)
Myo-inositol (77)	Progressive motility (↑); MDA (↓); total antioxidant capacity (↑); DNA damage (↓)
Resveratrol (28, 61–63)	DNA damage (↓); MDA levels (↓); SOD activity (↑)
Alpha-tocopherol and gamma-tocopherol (55)	Sperm viability and motility (↑)
*Opuntia ficus-indica* extracts (30)	DNA damage (↓)
Quercetin (43, 65)	Sperm motility and vitality (↑); DNA damage (↓); MDA levels (↓)

Overall, antioxidant supplementation was associated with reduced reactive oxygen species (ROS) levels, lipid peroxidation, DNA damage, apoptosis, and mitochondrial dysfunction, while improving sperm motility, viability, vitality, membrane integrity, and antioxidant capacity. Several antioxidants, including melatonin, MitoTEMPO, and resveratrol, also increased the activity of endogenous antioxidant enzymes such as superoxide dismutase (SOD), catalase, and glutathione peroxidase (GPx). BDNF, brain-derived neurotrophic factor; GSH, reduced glutathione; MDA, malondialdehyde; MMP, mitochondrial membrane potential; NADPH, reduced nicotinamide adenine dinucleotide phosphate; SOD, superoxide dismutase.

## Cryodamage, reactive oxygen species and sperm dysfunction

Cryopreservation is widely used in ART to preserve male fertility; however, the freezing and thawing process can significantly impair sperm molecular integrity and function (28, 29). Sperm survival after cryopreservation might be compromised, commonly referred to as cryodamage, and finally negatively affect sperm viability and motility (28, 29). During freezing, spermatozoa are exposed to several harmful conditions, including cold shock, osmotic stress, and ice-crystal formation. These factors can disrupt sperm physiology and membrane integrity. In particular, rapid temperature fluctuations may destabilize the plasma membrane and damage intracellular structures, reducing the fertilization potential of sperm cells. Cryoprotectants agents, although essential for limiting intracellular ice formation, may also contribute to cellular injury. Exposure to low temperatures and to cryoprotectants can alter the organization of cytoskeletal and membrane components, leading to modifications in sperm surface proteins that are essential for the sperm capacitation and sperm-oocyte interactions. Such alterations may impair capacitation, acrosome reaction, and overall fertilizing ability (15, 30, 31). Consequently, even spermatozoa that survive the freezing and thawing process may exhibit reduced functional competence. Recent advances in metabolomic and proteomic analysis have provided important insights into molecular pathways affected by cryopreservation. These analyses suggest that energy-related routes, including glycolysis, pyruvate metabolism, and the citrate cycle, are significantly altered during the cryopreservation and thawing procedures (32–34). Since sperm motility and fertilization capacity are highly dependent on energy production, disturbances in these pathways may contribute directly to reduced sperm performance after thawing. Furthermore, cryopreservation has been associated with changes in sperm gene expression profiles. Studies have demonstrated downregulation of messenger RNAs linked to fertilization, early embryo development, and successful pregnancy (35). Alterations in epigenetic-related transcripts have also been reported, suggesting that cryopreservation may influence not only sperm function but also post-fertilization developmental processes (35). Among the mechanisms involved in cryodamage, OS is considered one of the most important factors for reproductive and non-reproductive cells (13). OS occurs when the production of ROS exceeds the antioxidant defense capacity of the cell. During cryopreservation, OS may result from osmotic imbalance, membrane disruption, and increased oxidative metabolism during the thawing process. Reintroduction of oxygen to frozen tissue stimulates glucose-mediated oxidative metabolism, resulting in a sudden increase in ROS generation, commonly referred to as oxidative burst (36, 37). This process is mainly associated with dysfunction of the mitochondrial electron transport chain (38, 39). Mitochondria represent a major intracellular source of ROS in human spermatozoa, especially after cryopreservation (40, 41). Sperm cells contain limited cytoplasmic antioxidant defenses, and thus they are vulnerable to oxidative injury. Excessive ROS production can induce lipid peroxidation of sperm membranes, impair mitochondrial activity, and reduce ATP production, ultimately compromising sperm motility. OS generated during thawing has also been associated with increased DNA fragmentation (15). Several studies have identified ROS as primary mediators of DNA damage occurring during sperm freezing and thawing procedures (42, 43). Importantly, this DNA impairment appears to occur largely independently of caspase-related pathways, suggesting that oxidative mechanisms play a more direct role in cryoinjury (9, 42, 43). Ribas-Maynou and colleagues (15) demonstrated that cryopreservation significantly increases the proportion of spermatozoa with single-stranded DNA fragmentation, whereas no significant effect was observed on double-stranded DNA breaks. These findings indicate that OS preferentially induces specific forms of DNA damage during the cryopreservation process (15). The detrimental effect of OS may be even more pronounced in infertile or subfertile men. Increased levels of ROS after cryostorage have been associated with elevated sperm DNA fragmentation and oxidation in these patients. In addition, deficiencies in chromatin packaging by protamines, which are commonly observed in subfertile men, may render sperm DNA more vulnerable to ROS induced damage (15). Poor chromatin condensation reduces DNA stability and weakens protection against oxidative attack, thereby increasing the likelihood of genomic injury during cryopreservation. Overall, the extent of sperm DNA damage induced by cryopreservation appears to correlate closely with sperm dysfunction and male infertility (6). Since sperm DNA integrity is essential for normal fertilization, embryo development, and successful pregnancy outcomes, minimizing OS during freezing and thawing procedures remains a major objective in reproductive medicine. Continued research into antioxidant supplementation, optimized cryopreservation protocols, and improved cryoprotective agents may help reduce cryodamage and improve the clinical outcomes of sperm cryostorage.

## Antioxidants in human sperm cryopreservation

Although semen possesses intrinsic antioxidant defense systems, spermatozoa become increasingly vulnerable to procedure-induced OS during cryopreservation. This heightened vulnerability is largely attributed to the dilution of seminal plasma during sperm preparation and the addition of cryoprotective agents, both of which reduce the natural antioxidant capacity of the semen. Consequently, supplementation with external antioxidants has been proposed as a potential strategy to counterbalance the loss of endogenous protective mechanisms and minimize oxidative damage during freezing and thawing procedures (7, 44, 45). Enzymatic and non-enzymatic approaches have been widely investigated to reduce oxygen concentration, eliminate catalytic metal ions, scavenge superoxide and hydroxyl anions, as well as hydrogen peroxide, and limit membrane lipid peroxidation during cryopreservation. Several non-enzymatic antioxidants are capable of counteracting exogenous ROS and protecting sperm cells from OS induced damage (46). Vitamin E is a lipid-soluble antioxidant that, in its biologically active alpha-tocopherol form, scavenges superoxide anions, hydrogen peroxide, and hydroxyl radicals while interrupting lipid-peroxidation chain reactions within cellular membranes (47, 48). In addition, Vitamin E has been shown to reduce cryopreservation-related ROS generation and membrane lipid peroxidation, thereby preserving membrane stability and cellular function (49–54). Experimental studies in animal models demonstrated that vitamin E supplementation can improve post-thaw motility, viability, membrane and DNA integrity, while incubation with vitamin E during cryopreservation further enhances sperm quality and survival after thawing (25, 44, 50–54). An investigation conducted by Zerbinati and collaborators (55) evaluated *α*- and *γ*-tocopherol concentration in semen samples obtained from 134 men with different semen parameters using an isotope dilution gas chromatography–mass spectrometry method. The authors carried out freeze–thaw experiments to assess the protective effects of tocopherol supplementation during sperm cryopreservation. Their findings demonstrated that the addition of *γ*-tocopherol to cryopreservation media was associated with greater post-thaw sperm viability and motility compared with evaluated *α*-tocopherol supplementation, suggesting that γ-tocopherol may exert a stronger protective effect against OS and membrane damage induced by freezing and thawing procedures (55). Melatonin, a potent endogenous antioxidant secreted primarily by the pineal gland, also plays an important role in protecting sperm from OS (56). It has been identified in seminal plasma, and specific melatonin receptors are expressed on sperm membranes, supporting its physiological relevance in male reproduction (57, 58). Supplementation of freezing media with melatonin improves sperm viability and motility, as well as exerting cryoprotective effects by reducing OS during the freezing–thawing process. A study by Karimfar and co-authors investigated the effect of different concentrations of melatonin on semen samples collected from 43 fertile men. Samples were frozen and then thawed after 2 weeks. The authors reported that supplementation of freezing extender with melatonin, particularly at a concentration of 0.01 mM, improved post-thaw sperm viability and motility and decreased ROS and membrane malondialdehyde levels (29). Furthermore, the combined use of melatonin and caffeine has been associated with enhanced sperm motility and improved mitochondrial function after cryopreservation (59). Trans-resveratrol, a polyphenol compound naturally found in red wine and several plant-derived foods, exhibits antioxidant activity through free-radical scavenging and divalent-cation chelation (60). In addition to its antioxidant effects, resveratrol may inhibit ROS generation, promote calcium release into the cytoplasm, and increase phosphorylation of sperm adenosine monophosphate-activated protein kinase, thereby contributing to improved cellular metabolism and function (61). Resveratrol has also been shown to protect spermatozoa against ROS mediated lipid peroxidation and DNA damage, while increasing mitochondrial membrane potential and reducing intracellular ROS production (28, 61). However, despite these beneficial antioxidant properties, studies evaluating its role during sperm cryopreservation have produced inconsistent results, and resveratrol has not consistently demonstrated a protective effect on post-thaw sperm motility (62–64). The addition of ascorbic acid before cryopreservation has been shown to reduce sperm DNA damage, particularly in semen samples obtained from infertile men, suggesting a protective antioxidant effect against OS induced during the freezing–thawing process (63). Natural extracts and polyphenolic compounds have also been investigated for their potential protective effects during sperm cryopreservation. Extracts of *Opuntia ficus-indica*, which contain antioxidant molecules and flavonoids including resveratrol, were shown to reduce sperm DNA fragmentation, although no significant improvement in sperm motility or viability was observed (30). Quercetin supplementation has demonstrated beneficial effects by improving sperm motility and viability while reducing DNA damage and malondialdehyde levels, indicating decreased OS and lipid peroxidation (43, 65). In other biological systems, both resveratrol and quercetin have been reported to exert their antioxidant and cytoprotective effects through activation of sirtuin 1, deacetylation of liver kinase B1, and phosphorylation of adenosine monophosphate-activated protein kinase. However, whether these molecular pathways are involved in human sperm cryopreservation remains to be clarified (66, 67). Extracts derived from the brown alga Sargassum have also been investigated for their antioxidant properties during sperm cryopreservation. Sargassum extract, which contains polyphenolic compounds capable of chelating heavy metals and neutralizing free radicals, has been shown to improve both total and progressive sperm motility after the freezing–thawing process (68). Brain-derived neurotrophic factor (BDNF) is a neurotrophin-family polypeptide primarily expressed in the central nervous system, where it plays an important role in neuronal differentiation, maturation, survival, and regeneration (69). In the male reproductive system, BDNF is also expressed in Leydig and Sertoli cells, while its receptors have been identified in spermatogonia and mature spermatozoa, suggesting a potential role in the paracrine regulation of spermatogenesis (70, 71). In addition to its neuroprotective functions, BDNF may exert antioxidant effects by scavenging free radicals, modulating antioxidant enzyme activity, and increasing the expression of *sestrin 2*, a stress-responsive gene involved in cellular protection against oxidative damage (72, 73). Supplementation of freezing and thawing media with BDNF has been associated with improved sperm motility and viability. A study performed by Atefeh and collaborators, investigated the effect of BDNF supplementation on human sperm quality following the freezing and thawing process. Semen samples from 21 healthy fertile men were cryopreserved with or without BDNF, and post-thaw sperm motility, viability, ROS levels, caspase-3 activity, and AKT phosphorylation were assessed. The authors reported a significantly higher motility and viability in BDNF-treated samples, along with increased AKT phosphorylation. In addition, intracellular hydrogen peroxide levels and caspase-3 activity were significantly reduced. Overall, BDNF improved post-thaw sperm function by reducing OS and apoptosis, likely through activation of AKT signaling pathways (10). Other targeted antioxidant strategies investigated for sperm cryopreservation include glutathione, ascorbate, myo-inositol, and the mitochondria-targeted antioxidant MitoTEMPO. These compounds have been shown to improve post-thaw sperm motility, mitochondrial function, and antioxidant enzyme activity while reducing OS markers and DNA fragmentation (74–77). Elamipretide, a mitochondria-targeting peptide, has also demonstrated protective effects by improving post-thaw motility and viability, preserving the stability of the plasma membrane, mitochondria, and chromosomal structure, and reducing oxidative damage and acrosome dysfunction during the freezing–thawing process (78). In contrast, although L-carnitine supplementation enhances post-thaw sperm motility and vitality, it does not appear to provide significant protection against DNA oxidation (79). Additional protective approaches include the use of ganglioside micelles, which help preserve human sperm DNA integrity during freeze–thawing, reduce susceptibility to DNA fragmentation, modulate superoxide generation, inhibit iron-catalyzed hydroxyl-radical formation, and limit hydrogen peroxide diffusion across the sperm membrane, thereby contributing to reduced oxidative injury during cryopreservation (45). Finally, the addition of catalase to cryoprotective media has been shown to reduce ROS levels in frozen–thawed spermatozoa, improve sperm motility, viability and mitochondrial function, and inhibit DNA damage, as well as early apoptotic events associated with cryopreservation-induced OS (74). Although these findings are encouraging, no antioxidant strategy has yet been able to completely prevent cryodamage or fully preserve post-thaw sperm function. Several explanations have been proposed for this limited efficacy, including inadequate uptake or utilization of exogenous antioxidants by sperm cells, insufficient antioxidant concentration relative to the high levels of ROS generated during freezing and thawing, and progressive loss of antioxidant activity throughout the cryopreservation process. More recently, stress preconditioning before cryopreservation has emerged as a promising approach to stimulate intrinsic cellular defense mechanisms. Experimental studies suggest that exposure to sublethal hydrostatic pressure, osmotic stress, or controlled oxidative agents may enhance sperm tolerance to cryopreservation-induced injury and improve post-thaw sperm resilience (80–83).

## Conclusive remarks

Reactive oxygen species generation is considered one of the principal mechanisms underlying sperm cryodamage during cryopreservation. Human spermatozoa are particularly vulnerable to oxidative injury because they possess limited cytoplasmic antioxidant defenses and a plasma membrane rich in PFA, which is highly prone to lipid peroxidation. During the freezing and thawing process, excessive ROS production can impair several aspects of sperm physiology, including membrane integrity, mitochondrial activity, motility, viability, and genomic stability. OS may also induce DNA fragmentation and apoptosis-like changes, ultimately compromising the fertilizing potential of thawed spermatozoa. In routine sperm preparation procedures, seminal plasma is partially or completely removed prior to cryopreservation. Although this step is often necessary to improve sample quality and standardize freezing protocols, it simultaneously reduces the concentration of extracellular antioxidants naturally present in semen fluid, thereby increasing sperm vulnerability to OS. Consequently, considerable attention has been directed toward the development of protective strategies aimed at limiting ROS-mediated injury during cryopreservation. A large number of *in-vitro* studies have investigated the effects of supplementing freezing and/or thawing media with enzymatic and non-enzymatic antioxidants. These studies generally report a positive effect on post-thaw sperm parameters, including motility and viability, preservation of mitochondrial function and membrane integrity, and reduction in lipid peroxidation and DNA damage. However, the available evidence remains highly heterogeneous, mainly due to differences in study design, antioxidant type and concentration, cryopreservation protocols and cryoprotectants applied, and methods used to assess sperm quality. Importantly, no antioxidant strategy has yet proven capable of completely preventing cryodamage. Therefore, further well-designed human studies are required to define the optimal antioxidant molecules, effective concentrations, timing of administration, long-term safety, and overall clinical value of antioxidant supplementation in sperm cryopreservation media.
